# Understandings of spirituality and its role in illness recovery in persons with schizophrenia and mental-health professionals: a qualitative study

**DOI:** 10.1186/s12888-016-0796-7

**Published:** 2016-04-02

**Authors:** Rainbow Tin Hung Ho, Caitlin Kar Pui Chan, Phyllis Hau Yan Lo, Ping Ho Wong, Cecilia Lai Wan Chan, Pamela Pui Yu Leung, Eric Yu Hai Chen

**Affiliations:** Centre on Behavioral Health, The University of Hong Kong, 2/F, The Hong Kong Jockey Club Building for Interdisciplinary Research, 5 Sassoon Road, Pokfulam, Hong Kong; Department of Social Work and Social Administration, The University of Hong Kong, Pokfulam, Hong Kong; The Centre for Religious and Spirituality Education, Hong Kong Institute of Education, 10 Lo Ping Rd, Tai Po, Hong Kong; Department of Psychiatry, The University of Hong Kong, Pokfulam, Hong Kong

**Keywords:** Grounded theory, Holistic care, Mental-health professional, Qualitative methods, Persons with schizophrenia, Spirituality

## Abstract

**Background:**

Spirituality has received increased attention in the psychiatric literature; however, it remains underexplored on a global level. Knowledge about spirituality of persons with schizophrenia is often hampered by positive and negative symptoms, which limit their expression of spiritual needs and shift mental-health professionals’ focus from spiritual care to symptom control. Differences in the ways that the two parties understand spirituality may create different expectations and further hinder the provision of high-quality holistic care. This study investigated the meaning and roles of spirituality from the perspectives of persons with schizophrenia and mental-health professionals.

**Methods:**

A qualitative design with semi-structured individual interviews was adopted. The analysis was based on data collected from interviews with 18 clients diagnosed with schizophrenia and 19 mental-health professionals from public hospitals and mental-health community rehabilitation centres in Hong Kong. Data were collected and analysed based on grounded theory principles.

**Results:**

Both clients and professionals regarded spirituality as an inherent part of a person’s well-being, clients’ rehabilitation, and their lives in general. At the personal level, the clients’ descriptions were more factual, concrete, short term, and affective, whereas the professionals’ descriptions were more abstract, complex, and cognitive. At the communal level, both parties had a similar understanding of spirituality but different interpretations of its role in recovery from mental illness. The clients regarded spirituality as a source of giving and receiving love and care, whereas the professionals regarded it as a means of receiving support and managing symptoms.

**Conclusions:**

Building a common understanding on the concept of spirituality and the significant role it plays in rehabilitation between clients and mental-health professionals is an essential first step to support clients’ spiritual health. Clients tend to seek for stability, peace, and growth rather than an existential quest; while professionals hold a more pathological perspective, viewing spirituality as a means to relieve symptoms, increase social acceptance, and cope with illness experiences. The differential understanding of the two perspectives provides insight and perhaps a roadmap for developing spiritual assessments and holistic care in the psychiatric context.

## Background

In recent decades, increased attention has been devoted to psychosocial and spiritual care for mental-health rehabilitation [1]. This is, in part, because advances in pharmacological treatment have improved patients’ symptom control and functioning, which has allowed more time and opportunities to deal with other issues; some patients with mental disorders are discharged from the hospital and even reintegrate into the society. Thus, psychosocial and spiritual support, which can enhance patients’ overall functioning and quality of life, are important. While there has been a marked increase in studies generating evidence supporting psychosocial interventions, few studies in the international literature on psychiatric disorders (i.e. spirituality and mental-health care) have emphasised spiritual care in persons with psychotic disorders [2, 3].

There is documentation in the research literature that spirituality can help patients in the process of illness recovery by facilitating their autonomy and assisting them to live and grow beyond the limitations imposed by their illness [4]. Delgado noted that nurturing spirituality can increase one’s inner strength, personal awareness, and acceptance of the world, thereby enhancing the ability to cope with stress, uncertainty, and ambiguity, such as the sudden onset of an illness [5]. Several survey studies and meta-analyses have found that 30-90 % of people with schizophrenia or severe mental illnesses regarded spirituality or religion as one of the most essential resources for dealing with difficult/stressful events in the recovery process [6–10]. Most of them claimed that half of their time spent on coping was related to spiritual/religious practices [6].

However, the abstract and diverse conceptualisations of spirituality may impose barriers to the consideration of spiritual issues in clinical practice. Several studies have been conducted to explore mental-health professionals’ views about the role of spirituality in mental-health services. Crossley and Salter [11] interviewed a group of clinical psychologists to examine how they understood and addressed spirituality within the context of therapy. The study’s results indicated that spirituality was a diverse, difficult, and elusive concept to the clinical psychologists, although they noted that they would support their clients’ finding harmony through their spiritual beliefs. Barker and Floersch [12] revealed that social workers considered spirituality to be a nebulous and multidimensional concept. They used the following terms to describe spirituality: ‘metaphysical’, ‘beyond what can be known’, ‘humanly constructed’, and a phenomenon that ‘exists only because we experience and interpret it as such’ (p. 364) [12]. McSherry and Jamieson analysed the results of an online survey of nurses’ perceptions of spirituality and spiritual care [13]. Their findings suggested that nurses viewed spirituality as complex and multifaceted. To them, spirituality pertained to transcendent experiences as well as the values and experiences that the individual considered important, such as relationships, lifestyle, quality of life, and even the environment.

In addition, the spiritual dimension of life is often linked to psychopathology [14]. The psychotic symptoms of people with schizophrenia, such as hallucinations and delusions, may produce experiences that others consider bizarre and incomprehensible. Spiritual experiences, which are generally thought of as ‘special’ and ‘uncommon’ may be viewed as signs and symptoms of mental illness [15–18]. In a review of the relationship between schizophrenia and religion, Mohr and Huguelet reported that patients’ concerns about their psychiatrists’ views of their religious beliefs kept them from discussing religion with their psychiatrists, thereby eliminating a potential coping resource [19]. This situation revealed a lack of discussion and consensus on the understanding of spirituality between patients and mental-health professionals.

The existing literature rarely focuses on understanding spirituality from the perspectives of persons with schizophrenia [14], and discrepancies in the understanding of spirituality between people with schizophrenia and mental-health professionals have not been investigated. Possible gaps in understanding may cause different expectations in both parties, thereby hindering the provision and integration of spiritual support in long-term psychiatric care and mental-health services.

Thus far, few studies have focused on mental-health professionals’ understanding of their clients’ spirituality in relation to their illness. The findings of most previous studies have been based on the understanding of spirituality from the perspectives of professionals or relatively healthy populations (i.e. those functioning within normal limits), and then generalised to persons with schizophrenia. More importantly, even for the professionals, spirituality is recognised as a complex and difficult concept that may or may not be understood in the same way by persons with schizophrenia who do not have clear and logical thought processes from time to time. In light of this state of knowledge, questions about the possible roles that spirituality may play in the illness experience of schizophrenia, and whether persons with schizophrenia and mental-health professionals have different understandings of spirituality are remained to be answered. An investigation which, 1) allows persons with schizophrenia to speak about their experiences and interpretations of spirituality in relation to their illness, and 2) elicits mental-health professionals’ perspectives about their experiences and interpretations of spirituality in relation to the care that they provide, should contribute to a better understanding of the role that spirituality plays in the course of illness and recovery. This study was designed to explore how persons with schizophrenia and mental-health professionals understand spirituality and its roles in relation to illness experiences, and whether similar or different understandings exist.

## Methods

### Design

A qualitative design with a grounded theory approach was employed as it is necessary to explore the human experience when studying spirituality [20]. This strategy allowed us to explore systematically how the participants conceptualised and interpreted their personal experiences, without a presumed conceptual framework in their natural setting [21].

### Participants

Purposeful sampling [22] was used to recruit persons with schizophrenia (client group) and mental-health professionals (professional group) to yield focused, informative, and coherent findings on the topic of spirituality in schizophrenia. Eligible clients were referred by psychiatrists in the outpatient clinics of the Department of Psychiatry in one of the largest acute regional public hospitals of Hong Kong. All of the clients had a diagnosis of schizophrenia that was confirmed by a qualified psychiatrist, and they were receiving treatment for it. There were no established relationships between the interviewers and the participants prior to the study. The inclusion and exclusion criteria for the clients are shown in Table 1.

**Table 1:** Inclusion and exclusion criteria for persons with schizophrenia participated in the study

Inclusion criteria	
♦	Being diagnosed with schizophrenia according to the DSM-IV-TR by a psychiatrist
♦	Cognitive capacity to understand and provide informed consent to participate
♦	Ability to understand and communicate in Chinese
♦	Between the ages of 18 and 65 years
Exclusion criteria	
♦	Acute psychosis or neurological illness at the time of the interview
♦	Receiving any in-patient psychiatric care (hospitalisation)
♦	Diagnosed with co-morbid psychiatric disorders (e.g. major depression, personality disorders)
♦	Life-threatening medical illness that limits a client’s life expectancy to 1 year, as diagnosed by a physician
♦	Cognitive impairment as assessed by a mental-health professional that renders the client unable to participate in the interview

The mental-health professionals were recruited from 3 public hospitals, one of which was where the clients were recruited, and 3 non-governmental community mental-health rehabilitation centres. The main clientele that these clinical agencies served were persons with schizophrenia. The psychiatrists, nurses, occupational therapists, physiotherapists, and social workers were invited to participate in the study to maximise the range of perspectives that would be expected from practitioners of different disciplines.

The characteristics of the clients and professionals are shown in Table 2. The clients included 8 women and 10 men with a mean age of 28.4 years (*SD* = 5.3), ranging from 18 to 38 years. They had been diagnosed with schizophrenia for an average of 6.4 years (*SD* = 2.8). The professionals included 4 psychiatrists, 5 psychiatric nurses, 3 occupational therapists/physiotherapists, and 7 social workers. Eleven participants were women and 8 were men. The mean age was 38.83 years (*SD* = 6.2). The mean years of service in the hospital’s psychiatry department and rehabilitation institutions was 9.05 (*SD* = 9.4), ranging from 2 to 31 years.

**Table 2:** Participants’ characteristics

		Number	Percent
Persons with Schizophrenia		18	
Gender			
	Female	8	44.4
	Male	10	55.6
Education			
	Secondary	13	72.2
	College	1	5.6
	Bachelor’s or higher	4	22.2
Religion			
	None	10	55.6
	Christianity	5	27.7
	Buddhism	2	11.1
	Chinese folk religion	1	5.6
Mental-health Professionals		19	
Gender			
	Female	11	57.9
	Male	8	42.1
Occupation			
	Psychiatrist	4	21.0
	Nurse	5	26.3
	Occupational/physiotherapist	3	15.8
	Social worker	7	36.9
Religion			
	None	8	42.1
	Christianity	10	52.6
	Catholicism	1	5.3

### Data collection

Individual interviews were conducted at the outpatient clinics of the hospitals, the research centre of the university next to the hospital in which the clients were recruited, and the community rehabilitation centres, depending on the participants’ preferences and availability of the venue. Data were collected between February 2012 and September 2013. The participants’ demographic information, such as age, gender, educational level, denominational affiliation, and the number of years with the diagnosis (clients)/years of service (professionals) were collected at the beginning of the interview. All participants were asked to provide their written informed consent after the study’s purpose and procedures were explained to them. They also were assured that their personal data and identities would remain anonymous. Participant identification codes were used when reporting the contents of the interviews. Ethical approval was obtained from the Institutional Review Board (HKU/HA HKW) and the Research Ethics Committee (NTW) of the University of Hong Kong and the Hospital Authority.

### Interviews

Semi-structured interview guides were developed based on the relevant literature followed by consultations and discussions with the research team and mental-health professionals who were not participants of the study to ensure that the wording and sequence of the questions were clear and comprehensible to people with schizophrenia. Specific attention was paid to the use of language, as well as the sensitivity and appropriateness regarding culture-related issues.

The clients’ interviews began by inviting them to talk about their illness experiences rather than directly asking them about the meaning and experiences of spirituality. This is because spirituality is highly personal [23] and clients’ subjective experience have been found to be conceptualised in terms of their psychotic experience [24]. By talking about topics related to their illness experiences, the clients were easier to engage in the interview and shared their understanding of spirituality more readily. After talking about the meaning of spirituality, we invited them to talk about how spirituality related to their illness experiences. The professionals’ interviews started with direct questions related to their understanding of their clients’ spirituality. We also asked them what they thought about the role of spirituality in helping the clients to cope with and recover from their illness.

All of the interview questions were open-ended. Examples of questions included (1) Meaning of spirituality: ‘From your perspective, what is spirituality’? ‘What comes to your mind when you hear the term spirituality’? and ‘What do you think spirituality means to you’? (2) Composition of spirituality: ‘What makes up one’s spirituality’? and ‘What do you think spirituality includes’? (3) Spirituality in illness: ‘What is the role spirituality plays in your (clients’) illness experiences’? and ‘How do you think spirituality relates to your (clients) illness experiences’? The interviews were conducted in a manner that allows the participants to elaborate on the areas they considered interesting and relevant to spirituality.

Two researchers (PHYL and CKPC) conducted the interviews under the supervision of RTHH. Each interview took approximately 90 min and was terminated when the participant wanted to stop or when the questions in the four areas had been asked and no more elaborations were forthcoming. The conversations were audiotaped. Memos were taken during and after the interview in order to record the key issues that arose during the conversation. The interviewers paid close attention to clients’ mental and emotional states, although the clients were mentally stable and were prepared to report to on-site counsellors or physicians in the outpatient clinic if necessary. The first five interviews were conducted by the interviewers together in order to standardise the interview procedures and techniques. Then, they interviewed the participants separately.

### Data analysis

Data collection and analysis (coding and conceptualisation of data) is an iterative process. The analysis in this study followed the documented procedures of grounded theory [25–28], with Glaser’s approach which included the use of open and focused coding, research memos, and the technique of constant comparison. This approach allows concepts to emerge from the data [29, 30] rather than constructing framework through research practice [31], and putting data into preconceived framework [32]. Verbatim interviews were analysed separately for both groups and then compared using ATLAS.ti software. One of the research team members (CKPC) first became familiar with the data by reading the transcripts line-by-line. In vivo codes [28] using participants’ language/wording were generated to identify data showing participants’ understanding of spirituality and its role in illness experiences. Constant comparison and categorisation of codes were performed across and within the two groups of participants. The codes that were similar in their characteristics and nature were aggregated into subthemes and themes. No new concepts emerged from the fifteenth interview with clients and the sixteenth interview with the professionals. Three additional interviews were conducted in each group to ensure data saturation.

### Rigour

The trustworthiness of this study was ensured in several ways. First, an interview guide was developed before the study commenced to confirm the interviewers’ dependability during the process of data collection. Second, interviews were conducted until they reached a point of data saturation. Third, validation of the data was achieved by using triangulation in which both responses from persons with schizophrenia and mental-health professionals were obtained to confirm the validity of statements. Last but not least, the research team (RTHH, CKPC, and PHYL) also met regularly to discuss and resolve discrepancies in identifying, naming, and grouping of codes, subthemes, and themes. The analysis of transcriptions of each participant group involved two researchers who worked independently of each other. The percentages of agreement between the two researchers were 97.4 % for the client group and 85.2 % for the professional group.

## Results

Both the clients and professionals regarded spirituality as an inherent part of the clients’ well-being and rehabilitation, as well as their lives in general. However, the clients’ descriptions of spirituality were more factual (such as when they talked about their understandings of spirituality, they related it to self-knowledge which was about their concrete needs and thoughts); concrete and short term (when they described what they wanted to achieve in life); and affective (as they emphasised the calmness in heart when being disturbed by the psychotic symptoms, the pleasant transformation after acute phase of their illness, and the love and empathy manifested in religion). In contrast, the professionals’ descriptions were more abstract (such as they related spirituality to value or how much a person loves him/herself); complex (when they related spirituality to meaning in life and existential quest); and cognitive (as they identified changes after the acute phase of the illness as an analytical process of self-reflection and meaning making). As a whole, clients tended to seek stability, peace, and growth rather than an existential quest. The professionals had a more pathological perspective, viewing spirituality as a means to relieve symptoms, increase social acceptance, and cope with illness experiences. Based on the dimension of relatedness to oneself, analyses of the clients’ and professionals’ perspectives generated two major themes: (1) personal domain, and (2) communal domain. A summary of the major findings is presented in Table 3.

**Table 3:** Summary of the similarities and differences in perspectives on spirituality in two groups of participants

Meaning and Role of Spirituality in Illness			Persons with Schizophrenia	Mental-health Professionals
Personal domain	Sense of self	Meaning	Self-knowledge	Self-esteem
		Role	Knowing what to do in illness	Feeling Inferior in illness
	Philosophy of life	Meaning	Values, how to live a life	Meaning of life, existential quest
		Role	Concrete and short term goals	Purpose in life
	Growth	Meaning	Changes after acute episode	
		Role	Self-improvement	Meaning making
	Peacefulness	Meaning	Inner peace and calmness	
		Role	Coping with illness, stabilising symptoms	
Communal domain	Religion	Meaning	Religious affiliations and practices	
		Role	Love, empathy, knowing others	Social support, stabilising symptoms
	Interpersonal relationships	Meaning	Relationships with others	
		Role	As giver to help others	As care and support receiver
	Apparitional experiences	Meaning	Unusual experiences	
		Role	Just an experience	Delaying help seeking

### Personal domain

This theme illustrates one’s spiritual experiences in relation to elements on a personal level. Four subthemes were grouped under this domain: (1) sense of self, (2) philosophy of life, (3) growth after an acute phase of an illness, and (4) peacefulness. The meanings of three of the four subthemes and their roles in illness recovery were described differently by the clients and professionals.

1. Sense of self
  Sense of self was identified as a component of spirituality by both the clients and professionals. It referred to a person’s conceptual image and perception of himself/herself.
  ‘[How spirituality relates to my mental well-being]…I don’t have a clear image about myself’. (Client 012)
  ‘Spirituality refers to how a person perceives the value of him/herself and also one’s self-image’. (Professional 002)
  However, the clients emphasised how much they know about themselves (i.e. self-knowledge) and tended to associate sense of self with an understanding of their thoughts and subjective feelings/emotions. The professionals related sense of self to how much a person loves him/herself (i.e. self-esteem and recognition of the value of oneself).
  ‘Spirituality means being sensible… Basically, during the onset of my illness, I didn’t know what I needed to do, and what I wanted to do… I felt like… I didn’t have any thoughts or feelings’. (Client 011)
  ‘Spirituality is talking about how to appreciate one's own self.’ (Professional 004)
2. Philosophy of life
  Both clients and professionals associated spirituality with some aspects of their philosophy of life, which included personal beliefs/values and internal attitudes. Clients seldom talked about their long-term goals but they mentioned short-term and concrete goals, such as seeking stability in their lives especially during and after an illness episode. Professionals, on the other hand, related spirituality to meaning in life, which they thought should include the quest for existence.
  ‘Spirituality may relate to one’s internal attitudes (values)…that is, how you treat your life…and how you live your life’. (Client 002)
  ‘[Interviewer: When we talked about spirituality, you said you want to have some purposes in your life. Is there anything you want to achieve in life?] I don’t have any because I think it sounds too far away… (I want to) graduate from University first’. (Client 001)
  ‘I think it refers to… how the person views his/her life. That is, talking about meaning in life, one’s own existence… it’s not just about gratifying the hunger instinct’. (Professional 008)
  ‘Through human suffering, he/she would experience lots of bitterness and negative feelings. All these are related to the existential issue which represents the aspect of spirituality’. (Professional 005)
3. Growth after the acute phase of an illness
  Both groups of participants mentioned changes in clients who had gone through an acute phase of their illness. The clients’ descriptions focused more on their ongoing personal growth and improvement. Some clients even described the changes as a pleasant ‘transformation’. The professionals, who used clinical terminology, identified such changes as an analytical process of self-reflection and ‘meaning making’.
  ‘Spirituality is constantly improving, enhancing, and changing oneself’. (Client 002)
  ‘When I look back, my illness means a lot to me [spiritually]… I am more cheerful now. In the past, I kept everything secret because I was afraid of being teased and was feeling insecure… I was so scared that they would look down on me… But I’m no longer like this. I’m no longer afraid of being looked down on and teased by others. I’m fearless now’. (Client 011)
  ‘Spirituality refers to how a person reviews his/her life… but for the patients… the illness affects the patient’s most fundamental aspects, such as his cognitive functions and how he perceives the authenticity of the external world. So when a patient is experiencing an acute episode, he is in a chaotic condition. When he is getting better, he will try to explain what happened before, what had gone wrong, how to explain those things, or how to cope with his current situation’. (Professional 003)
4. Peacefulness
  Peacefulness was the only component of spirituality in this domain that was interpreted in the same way by clients and professionals. They both viewed peacefulness as having stability in life that could help them keep calm, stabilise symptoms, and cope with chaos during an acute episode of their disease and recovery. Clients, in particular, emphasised the role of peacefulness (e.g. the calmness in heart induced by the sense of peacefulness) in helping them cope with illness experiences, more than the professionals did. A more detailed account of this role has been reported in which clients mentioned that a sense of peace could help them break the vicious cycle of intensifying irrational thoughts and feelings during an illness episode (Chan et al., 2015).
  ‘(Spirituality) refers to a sense of peace, stability, and tranquility… a moment of staying calm’. (Client 024)
  ‘Spirituality is a sense of peace… like those people who can stay calm when facing troubles… I once attempted to jump from a building… at that time my mental state was extremely chaotic… At the very last minute, I thought of God [a place she found peace]… (My entire being) got settled down [calmed down]. All the chaos stopped, and then someone came and pulled me back into the house’. (Client 001)
  ‘Spirituality refers to the pursuit of inner peace, which means stability in one’s life’. (Professional 003)
  ‘How patients understand spirituality? … I think they are hoping to have a peaceful and stable life. It means no more symptoms or readmissions to the hospital due to the illness… not being annoyed by the hallucinations. In fact, lots of patients with schizophrenia indicated that when they were able to get rid of all the symptoms, to them, it already represented a high quality of life’. (Professional 008)

### Communal domain

This domain illustrates one’s spiritual experiences in relation to people and their surrounding systems/affiliations, as well as supernatural entities (i.e. beyond the personal level). Three categories were grouped under this domain: (1) religion, (2) interpersonal relationships, and (3) apparitional experiences. The meanings of these categories were similarly understood by both participant groups, but their roles in illness recovery were described differently.

1. Religion
  Both clients and professionals, regardless of their religious affiliations, associated spirituality with religion, which was understood as a range of religious denominations, activities, and beliefs. Yet, its role in illness recovery was viewed differently by the two groups. For the clients, spirituality represented love and empathy when it was manifested in religion. It not only represented the ability of a higher power to empathise with the client, but it also represented the client’s acquired ability to empathise with others. It enabled the clients to feel connected. From the point of view of the professionals, relating spirituality to religion might help clients improve their social support system, thereby, stabilising their mind and some of their symptoms.
  ‘Maybe God really represents love. This may sound perfunctory to other people, but He truly represents love. He is empathetic. It doesn’t mean that He can feel what I feel when I am suffering. It means that when I see others suffering, [His empathy and love] make me understand how they feel so that I am more willing to help them’. (Client 002)
  ‘For religion… at least patients can have a group to support them. I think the families of most patients may not be able to provide sufficient support to them because they may not have adequate knowledge of the illness, and may even blame the patients for some of their behaviors. But people in a religious organisation, are willing to support the patients unconditionally and have more time to listen to their stories, so that the patients can obtain psychological or spiritual support’. (Professional 009)
2. Interpersonal relationships
  For both clients and professionals, spirituality represented the connections and social interactions between clients and other people. However, clients viewed spirituality as a component of a mutual relationship in which they realised their capacity to influence others, and be influenced by them. The professionals regarded the clients as mere recipients of a relationship in which they could benefit from being cared for, loved, and accepted throughout their recovery.
  ‘Regarding my spirituality, I see that everyone is changing. In fact, it’s life affecting life; others influence me and I influence them as well’. (Client 002)
  ‘Spiritually, what makes patients feel touched…? I think it’s the care and love in social relationships… They’d sometimes tell me that they really appreciate my willingness to listen to them or just accept their silence… Having trust in the relationship between patients and professionals makes the illness more manageable when patients relapse because they think that I’m here to help them’. (Professional 008)
3. Apparitional experiences
  Both clients and professionals described apparitional experiences as part of the clients’ spirituality. They referred to unusual experiences, such as being possessed or seeing something that was usually regarded as unreal or extraordinary. However, only professionals mentioned the negative effects of apparitional experiences on illness recovery, such as delayed treatment and medication non-adherence.
  ‘Spirituality… it’s easy for me to see those things [spirits of the dead]. Just like my dad, who has already passed away, I can see him when he comes back to visit me. I’ve also seen my grandpa’. (Client 005)
  ‘From a traditional Chinese perspective, it is seen as being possessed by a demon or being able to see evil spirits. Patients’ family members would go and ask for some talismans and charms for them to eat… Such behaviors always delay their help seeking… even if they sought help, they might not believe in you. They’d think there might be other possibilities [that could cure the illness], and thus, refuse to take the medicine’. (Professional 004)

## Discussion

### Major findings

Seven subthemes of spirituality in the personal and communal domains were identified in both the client and professional groups. The data showed some overlapping in the interpretations of each subtheme between the two groups, but differences still existed. Spirituality was commonly understood as an essential part of human beings as well as connectedness to others in the external world. The only subtheme that both groups had a similar understanding of was peacefulness. Different views about the meaning and roles of spirituality were observed in the subthemes of *sense of self*, *philosophy of life*, and *growth after an acute phase of an illness*. Both groups had similar interpretations of the meanings of all the other subthemes of spirituality, but had different views of the roles that the subthemes played in illness recovery.

The conception of spirituality that emerged in this study echoes the contemporary definitions of spirituality. Despite differences in terminology, both the present findings and previous works regard spirituality as ‘an inherent component of being human’, and ‘subjective, intangible, and multidimensional’ [33]; it is ‘a quality of a human being that is not reducible to any part’ [34]. The descriptions of spirituality by clients and professionals were similar to the interpretations of spirituality in the literature [5, 35, 36], such as the capacity to be conscious of oneself, to achieve peacefulness and growth despite illness-induced limitations, and to have a belief system, with cardinal values guiding thoughts and actions (i.e. a life philosophy). The relationship to self, other persons, religion, and the supernatural components mentioned by the participants also reflected the concept of ‘harmonious interconnectedness between self, others/nature, and the Ultimate Other, which exists throughout and beyond time and space’, as described by Hungelmann and colleagues (p. 263) [37].

Furthermore, the findings indicated that both the clients and professionals recognised the importance of clients’ experiences of connectedness in their illness recovery. The more connected a client is with him/herself, others, the physical world, and a higher power, the more spiritual the client is, thus, the more likely the client will be to have a better recovery. This is in line with Eberst’s idea that the experience of connectedness relates closely to one’s richness of spirituality and holistic well-being [38].

### Major discrepancies between the clients and professionals

#### Giver and taker of intrinsic and extrinsic needs

At the communal level, clients and professionals understood the components of spirituality in a similar way, but perceived the roles they played in illness recovery in different ways. Clients tended to regard the connections to others and religion as sources of fulfilment of their intrinsic needs for love, care, and acceptance. Some of them even viewed themselves as providers who could use their experiences to help others. Professionals, on the other hand, regarded these connections as more functional, such that clients could obtain social support from others, which in turn could help stabilise their mind and symptoms. However, when the connections were perceived as apparitional experiences, it could be a hindrance to early treatment and medication adherence. Professionals also regarded clients as recipients and beneficiaries. These discrepancies may be due to the role and training that professionals have in psychiatric settings. Professionals tended to regard clients’ different resources as a means to help ‘fix’ the clients’ malfunctions, rather than the clients’ desires for the most fundamental and essential human needs. Clients, not the mental-health providers, should be the recipients of help and care.

#### Characteristics of schizophrenia

At the personal level, however, the understanding of spirituality was found to be very different in clients and professionals. Even when prompted, clients’ descriptions of spirituality were more factual (understanding of one’s thoughts and feelings) and present-moment oriented (focusing on continuing personal growth). They tended to associate spirituality with concrete and short-term goals and affective experiences (pleasant transformation and peace), rather than the search for one’s place in the universe, one’s meaning/purpose in life, or the ultimate quest for one’s existence and nonexistence, which are consistently mentioned in contemporary definitions [5, 36, 39]. In contrast, the professionals’ descriptions were more abstract, complex, and cognitive (i.e. self-esteem, self-reflection, meaning in life and meaning making). This difference was most likely due to the characteristics of schizophrenia. First, the cognitive deficits in schizophrenia interfere with the persons’ executive functioning [40], which includes problem-solving skills, planning, abstract/inferential thinking, and the ability to access stored knowledge and strategies [40, 41]. Research shows that these skills are crucial prerequisites for engaging in meaningful activities and redefining lives [40]. Therefore, the participants in this study with schizophrenia may not have had the cognitive competence to engage in abstract thinking, make meaning of their illness, or reflect on issues of meaning/purpose in life or existential quests. Second, meaning and purpose in life and the search for one’s existence seem especially important to those facing functional deterioration and approaching death because they permit a new perspective on the self and others, time and space, and life and death [42], enabling the individual to accept death and disease as a natural part of life. Schizophrenia, however, is neither a physical deterioration nor a life-threatening disease. Therefore, it would not be expected to induce an urge in the participants to review their lives and their contributions to others and society. Third, the positive psychotic symptoms create disturbances in emotions and thinking [41], influencing the affected individuals’ ability to communicate, work, and live as ordinary people. Therefore, staying calm and clear-minded to allow themselves to sense, feel, and judge is more meaningful to them and thus, becomes their main concern.

#### Cultural influences

The conceptualisation of spirituality observed in this study may partly reflect cultural influences. Buddhism has a long history in the Chinese culture. It emphasises peace of mind and is often associated with the practice of meditation and mindfulness, which is considered the ‘heart’ of Buddhism [43]. Though Hong Kong is considered a ‘westernised’ international city, with a history of being governed by the British government for 157 years, the influence of traditional Eastern culture cannot be ignored. In this analysis, both clients and professionals mentioned peacefulness when they interpreted the meaning of spirituality, regardless of their religious backgrounds. This might reflect the embedded cardinal value of emphasising harmony, calmness, and inner peace in the Chinese culture. For clients in particular, an inner sense of peace might help them moderate their level of distress and cognitive disturbances and enable them to cope better with their illness [44].

In addition, none of the clients and professionals mentioned about the relationship between spirituality and the onset of schizophrenia. This phenomenon may be explained by the way Chinese people define mental illness. In Hong Kong, people often use the term “chi xian” to describe persons who are mentally ill. This term literally means short-circuited in the brain which implies neurological abnormalities inside the brain [45], but not about any aspects of spirituality. Many Chinese lay beliefs also consider mental illness as a result of one’s moral lapse [46], transgression in this/previous life [47, 48], weak character [49], and irreverence for ancestors [50]. These beliefs are often found to be different from the ideas presented in the existing literature regarding the relationship between spirituality and the onset of mental illness, which are more about mystical (spiritual) experiences and spiritual possession in psychotic experiences [51–53].

### Limitations

The limitations of this study include the presence of inclusion and exclusion criteria for the recruitment of persons with schizophrenia, the cultural exclusivity of the sample, and the verbal nature of the data collection method. First, the sample was restricted by the selection criteria, and thus, caution must be taken in generalising the results to people with schizophrenia and co-morbidities. Second, the sample consisted of Chinese outpatients in a hospital. Although Hong Kong is an international city in which people are very westernised, caution should be exercised when generalising the findings to people with schizophrenia of other races and cultures and to those who have been institutionalised for a long period. Future studies should consider recruiting participants from different cultures and religious orientations to make comparisons possible. Third, this investigation is limited by the researchers’ background in which they were primarily trained as mental-health researchers rather than theologians or religious scholars. This may limit the interpretation of data. For example, the terms used to describe the concept of spirituality may be overly general and not theologically/religiously/spiritually sounded. Furthermore, a categorical approach was used to understand spirituality, rather than viewing spirituality in different levels of depth. Finally, spirituality is not easy to articulate, and it requires a person to have relevant knowledge and language skills. The verbal nature of the investigation may have prevented those who were not verbally expressive or articulate from fully expressing their views, even if they were very spiritual. These limitations indicate the importance of developing a multi-method research approach, which includes verbal and non-verbal means of communication and expression.

## Conclusion

The present study revealed similarities and differences between persons with schizophrenia and mental-health professionals in their understandings of spirituality and its roles in illness recovery. Persons with schizophrenia might not use abstract concepts, such as meaning in life and existential quest because of cognitive deficits. They might not need to confront an existential crisis. However, they are better at understanding factual and concrete knowledge, and interested in sensing and feeling their lives. They want to be the givers rather than only receivers of support, in contrast to what the professionals expected them to be. These findings suggest that the use of a strength- and ability-focused perspective rather than a weakness- and symptom-focused perspective in interventions may benefit clients. In addition, the operational definition of spirituality should go beyond the areas of meaning in life and existential quest when conducting research on spirituality with populations of people with schizophrenia, in order to prevent biased results. Research methods that rely less on cognitive function and verbal articulation should also be used in future studies. Finally, the relationship among the experiences of connectedness, richness of spirituality, and holistic well-being, as reported by the participants, indicate the value and feasibility of conducting spiritual interventions for this population, with prioritised goals. Interventions that can help persons with schizophrenia foster a stable and peaceful mind and improve their sense of connectedness to the self and others should be particularly useful.

### Ethics

Ethical approval was obtained from the Institutional Review Board (HKU/HA HKW) and the Research Ethics Committee (NTW) of the University of Hong Kong and the Hospital Authority.

### Consent to participate and consent to publish

All participants signed a form of consent to participation and to publication of interview contents before participation. They were assured that their personal data and identities would remain anonymous.

### Availability of data and materials

By contact with corresponding author.

## Abbreviations

HKU/HA HKW: The University of Hong Kong and the Hospital Authority Hong Kong West Cluster
NTW: New Territories West Cluster
SD: standard deviation
